# Circadian Differences in the Contribution of the Brain Renin-Angiotensin System in Genetically Hypertensive Mice

**DOI:** 10.3389/fphys.2018.00231

**Published:** 2018-03-19

**Authors:** Kristy L. Jackson, Francine Z. Marques, Kyungjoon Lim, Pamela J. Davern, Geoffrey A. Head

**Affiliations:** ^1^Neuropharmacology Laboratory, Baker Heart and Diabetes Research Institute, Melbourne, VIC, Australia; ^2^Department of Pharmacology, Monash University, Victoria, VIC, Australia; ^3^Heart Failure Research Group, Baker Heart and Diabetes Research Institute, Melbourne, VIC, Australia; ^4^Department of Physiology, Anatomy and Microbiology, Latrobe University, Bundoora, VIC, Australia

**Keywords:** renin angiotensin system, angiotensin II, neurogenic hypertension, BPH/2J mice, central nervous system, reactive oxygen species

## Abstract

**Objective:** Genetically hypertensive BPH/2J mice are recognized as a neurogenic model of hypertension, primarily based on sympathetic overactivity and greater neuronal activity in cardiovascular regulatory brain regions. Greater activity of the central renin angiotensin system (RAS) and reactive oxygen species (ROS) reportedly contribute to other models of hypertension. Importantly the peripheral RAS contributes to the hypertension in BPH/2J mice, predominantly during the dark period of the 24 h light cycle. The aim of the present study was to determine whether central AT_1_ receptor stimulation and the associated ROS signaling contribute to hypertension in BPH/2J mice in a circadian dependent manner.

**Methods:** Blood pressure (BP) was measured in BPH/2J and normotensive BPN/3J mice (*n* = 7–8) via pre-implanted telemetry devices. Acute intracerebroventricular (ICV) microinjections of AT_1_ receptor antagonist, candesartan, and the superoxide dismutase (SOD) mimetic, tempol, were administered during the dark and light period of the 24 h light cycle via a pre-implanted ICV guide cannula. In separate mice, the BP effect of ICV infusion of the AT_1_ receptor antagonist losartan for 7 days was compared with subcutaneous infusion to determine the contribution of the central RAS to hypertension in BPH/2J mice.

**Results:** Candesartan administered ICV during the dark period induced depressor responses which were 40% smaller in BPH/2J than BPN/3J mice (*P*_strain_ < 0.05), suggesting AT_1_ receptor stimulation may contribute less to BP maintenance in BPH/2J mice. During the light period candesartan had minimal effect on BP in either strain. ICV tempol had comparable effects on BP between strains during the light and dark period (*P*_strain_ > 0.08), suggesting ROS signaling is also not contributing to the hypertension in BPH/2J mice. Chronic ICV administration of losartan (22 nmol/h) had minimal effect on BPN/3J mice. By contrast in BPH/2J mice, both ICV and subcutaneously administered losartan induced similar hypotensive responses (−12.1 ± 1.8 vs. −14.7 ± 1.8 mmHg, *P*_route_ = 0.31).

**Conclusion:** While central effects of peripheral losartan cannot be excluded, we suggest the hypotensive effect of chronic ICV losartan was likely peripherally mediated. Thus, based on both acute and chronic AT_1_ receptor inhibition and acute ROS inhibition, our findings suggest that greater activation of central AT_1_ receptors or ROS are unlikely to be mediating the hypertension in BPH/2J mice.

## Introduction

BPH/2J mice are a genetic model of hypertension selectively bred from the same base population as their normotensive BPN/3J control strain (Schlager, 1974). BPH/2J mice have a neurogenic form of hypertension primarily based on the finding that ganglion blockade reduces BP to comparable levels in BPH/2J and BPN/3J mice (Davern et al., 2009). The contribution of the sympathetic nervous system (SNS) to the maintenance of blood pressure (BP) has recently been demonstrated to be nearly 2-fold greater in BPH/2J than BPN/3J mice, during both the light and dark period, suggesting a tonic over-activation of the SNS (Jackson et al., 2013). There is also growing evidence that the central nervous system (CNS) may be involved in the hypertension in this model. Early studies report that whole brain catecholamine levels were low in BPH/2J mice and more discrete evaluation revealed that catecholamine levels were particularly low in the hypothalamus, midbrain, medulla, and thoracic spinal cord of BPH/2J mice (Schlager and Freeman, 1983; Schlager et al., 1983; Denoroy et al., 1985). Whether these differences in catecholamine level were due to lower synthesis or increased turnover is unknown but it does highlight a difference in the CNS that is specific to the hypertensive mice. Brain-imaging studies also showed differences in neuronal activity in key autonomic cardiovascular regulatory brain regions using both cytochrome oxidase and Fos as markers of neuronal activity (Strazielle et al., 2004; Davern et al., 2009). Most recently, we have shown that neuronal overactivity in the medial amygdala is a major contributor to the sympathetically mediated hypertension in BPH/2J mice (Jackson et al., 2014). Taken together these studies suggest that the CNS is likely involved in the sympathetically mediated hypertension in BPH/2J mice, but the mechanism remains to be determined.

In the CNS, angiotensin II (AngII) is an important neuromodulator which regulates the cardiovascular system via modulating sympathetic vasomotor tone, vasopressin release and dipsogenic and sodium appetitive responses (Keil et al., 1975; Head, 1996; Fitzsimons, 1998). The AT_1_ receptor is the predominant receptor subtype in the CNS which mediates the stimulatory actions of AngII on BP (McKinley et al., 1996; von Bohlen und Halbach and Albrecht, 2006) although there is some evidence that stimulation of central AT_2_R can also influence BP (Li et al., 2003). Furthermore, reactive oxygen species (ROS) derived from nicotinamide adenine dinucleotide phosphate (NADPH) oxidase has been shown to mediate some of the cardiovascular actions of AngII in the CNS (Zimmerman et al., 2002; Chan et al., 2005). Importantly, the central renin angiotensin system (RAS) appears to play a critical role in a range of models of hypertension including models which are shown to have sympathetically mediated hypertension (Campese et al., 2000; Ito et al., 2002; Sun et al., 2002; Ye et al., 2002).

The contribution of the RAS to hypertension in BPH/2J mice is unclear because of conflicting reports in the literature (Leckie, 2001; Palma-Rigo et al., 2011). The apparent inconsistency in the contribution of the RAS to hypertension in BPH/2J mice has been speculated to relate to variations over the 24 h period as well as different contributions from the central and peripheral RAS (Jackson et al., 2013). Indeed our recent findings show that the peripheral RAS contributes to hypertension specifically during the dark (active) period in BPH/2J mice. Treatment with the ACE inhibitor enalaprilat, which does not readily cross the blood-brain barrier (BBB), caused a 4-fold greater depressor response in BPH/2J than BPN/3J mice (Jackson et al., 2013). Based on the overactivity of the peripheral RAS in BPH/2J mice, it is also possible that the central RAS is more active in BPH/2J mice.

We hypothesize that greater AngII stimulation of AT_1_ receptors and subsequent ROS signaling in the brain may contribute to the hypertension and exaggerated cardiovascular responsiveness to stress in BPH/2J mice. The aim of the present study was to pharmacologically assess the contribution of the central RAS to the hypertension in BPH/2J mice using acute and chronic intracerebroventricular (ICV) administration of AT_1_ receptor antagonists. Additionally, we examined the contribution of central ROS to hypertension in BPH/2J mice, using acute ICV microinjection of the superoxide dismutase (SOD) mimetic tempol and the ROS scavenger resveratrol. Furthermore, the cardiovascular effects of these drugs were assessed during the light and dark periods of the 24 h light cycle and during stress, to assess the contribution during different states of arousal. Finally, we further analyzed our previously published genome-wide gene array (Marques et al., 2011a), to determine the hypothalamic expression of RAS related mRNA including angiotensinogen, enzymes involved in the production of angiotensin (renin, ACE) and angiotensin receptors (AT_1a_, AT_1b_, AT_2_, MAS).

## Methods

### Animals

Experiments were performed on hypertensive BPH/2J (*n* = 26) and normotensive BPN/3J (*n* = 26) age-matched, adult male mice. The mice were housed individually in a room with 12:12 h light-dark cycle (1 a.m.–1 p.m. light) and allowed access *ad libitum* to water and mouse chow (Specialty Feeds, Glen Forrest, Western Australia, 19% protein, 5% fat, 5% fiber, 0.2% sodium). This study was carried out in accordance with the recommendations of the Australian code for the care and use of animals for scientific purposes, National Health and Medical Research Council. The protocol was approved by the Alfred Medical Research Education Precinct Animal Ethics Committee.

### ICV guide cannula implantation

ICV guide cannulae were implanted under a Ketamine (Ketalar, Pfizer)/Xylazine (Ilium Xylazil-20, Smithfield, Australia)/Atropine (Sigma, St. Louis, USA) mixture, 100, 10, and 1.2 mg/kg respectively. A computer aided stereotaxic apparatus (Angle Two Mouse, Leica) was used to position the guide cannula 0.5 mm posterior from bregma, 1.2 mm lateral from the midline, and 2.2 mm ventral to the skull surface. The guide cannula was secured in place with M 1.0 cheese-head screws (Mirofasteners, Melbourne) and dental cement (Vertex, Zeist, The Netherlands). The guide cannula used for the acute study were 26G with 33G injector (Plastics One). Right angle cannula (30G) for chronic ICV administration of drugs, were attached to SP10 tubing via SP45 tubing which was tunneled along the back of the neck for connection to a minipump at a later time. Anesthesia was reversed with 0.2 mg/kg Atipamezole HCl (Antisedan, Pfizer). Mice were allowed 14 days to recover prior to subsequent BP telemetry probe implantation surgery.

### Telemetry probe implantation

BP telemetry transmitters (model TA11PA-C10; Data Sciences International, St Paul, Minnesota, USA) were implanted under isoflurane open circuit anesthesia (5% induction and 1.5–2% maintenance; Forthane, Abbott, Botany, Australia). The catheter of the telemetry device was inserted into the carotid artery and the transmitter probe was positioned subcutaneously along the right flank (Butz and Davisson, 2001). Post-operative analgesia was provided by subcutaneous (SC) administration of 5 mg/kg Carprofen (Rimadyl, Pfizer Australia Pty Ltd., West Ryde, NSW, Australia).

#### Cardiovascular and locomotor activity measurements

After a 10 day recovery period, a baseline 48 h recording of systolic (SAP), diastolic (DAP), and calculated mean arterial pressure (MAP), heart rate (HR), and locomotor activity were measured in freely moving mice in their home cage. The recordings were sampled at 1,000 Hz using an analog-to-digital data acquisition card (National Instruments 6024E) as described previously (Jackson et al., 2007).

### Protocol for acute cardiovascular response to drugs

The effect of each drug on cardiovascular parameters was determined during the dark (active) and light (inactive) period of the 24 h light cycle. Cardiovascular parameters were measured 30 min before and 30 min following ICV injections (1 μl) of AT_1_ receptor antagonist, candesartan (5 nmol); SOD mimetic, tempol (2 μmol; Sigma-Aldrich, NSW, Australia), and ROS scavenger, resveratrol (1 nmol; Tocris biosciences, Bristol, UK). In separate mice, the acute cardiovascular response induced by ICV AngII (30 ng in 1 μl) was also measured for 10 min in BPN/3J and BPH/2J mice. Doses of drugs used in the present study were based on acute pilot dose studies which identified doses that induced cardiovascular effects in these mice. Acute ICV injections were performed on separate days with a minimum of 48 h recovery before a subsequent injection was administered during either light or dark. The sequence of drugs was assigned according to a Latin square design, thus there was not a fixed order and treatment order was different between mice. The acute ICV injections involved disturbing and handling the conscious mice, therefore a sham control group was included to demonstrate the effect of this procedure without any ICV injection. Furthermore the cardiovascular effects of the drugs were measured from 15 to 30 min after the ICV injection to minimize the influence of this handling on cardiovascular parameters. Tempol was dissolved in Ringer's solution (Baxter, NSW, Australia), candesartan stock solution was dissolved in 1 M Na_2_CO_3_ and diluted in Ringer's solution and resveratrol was dissolved in 5% DMSO in Ringer's solution. All drugs were freshly prepared each day.

Restraint stress induced cardiovascular changes were determined during the light (inactive) period 30 min following ICV injection of drugs. Mice were restrained in a flexible transparent conical shaped plastic restrainer (Decapicone, Braintree Scientific, USA) for a period of 5 min.

### Protocol for chronic ICV administration of losartan

#### Preliminary dose finding study

For chronic inhibition of AT_1_ receptors, losartan was used as it was more readily available than the candesartan used in the acute study. To identify an effective central dose with minimal peripheral effects, a preliminary acute ICV dose finding study for losartan (0.4, 2, and 10 μg) was performed on separate days in BPN/3J and BPH/2J mice (*n* = 3/group) and compared with an equivalent dose delivered subcutaneously. The dose of losartan selected for chronic infusion was chosen to mimic a similar steady state response as the depressor response to an ICV bolus of 10 μg (22 nmol) losartan.

#### Main chronic study protocol

Following 48 h of baseline cardiovascular and locomotor activity measurement, mice underwent a series of baseline behavioral tests detailed below. Each mouse received infusion (0.22 μl/h) of either losartan (22 nmol/h) or Ringer's solution (Baxter, NSW Australia) via both the ICV and SC routes, in a crossover design. Minipumps (Alzet, model 1002) filled with either losartan or Ringer's solution, were implanted subcutaneously through a small incision between the scapula, under isoflurane open circuit anesthesia (5% induction and 1.5–2% maintenance). For ICV infusion, minipumps were connected to pre-implanted ICV guide cannula via SP45 tubing. Following 7 days of treatment, cardiovascular parameters, and locomotor activity were recorded for 48 h and the behavioral tests were subsequently repeated. Mice then received the treatment by the alternate route of administration. Following 7 days of treatment, cardiovascular parameters and locomotor activity were recorded for another 48 h and the behavioral tests were subsequently repeated.

### Behavioral tests

Mice were exposed to two different aversive behavioral stresses performed on separate days during the light period when the animals were inactive (Davern et al., 2009, 2010). Restraint involved guiding the mouse into a cylindrical plexiglass restrainer with a sliding back plate to confine the animal for 60 min. Dirty cage-switch stress involved removing the mouse from its home cage and placing for 60 min in a cage previously occupied by another male mouse. MAP responses were analyzed as changes from baseline as described previously (Davern et al., 2010).

### Analysis of gene array data from the gene expression omnibus repository

Expression of genes in the renin-angiotensin system (measured as mRNA) was determined based on a previously published (by us) transcriptome-wide gene array available in the Gene Expression Omnibus (GEO) repository (located at https://www.ncbi.nlm.nih.gov/geo/query/acc.cgi?acc=GSE26007) (Marques et al., 2011a). Briefly, this gene array data details mRNA abundance in hypothalamic tissue of adult BPN/3J and BPH/2J mice, collected during the light (*n* = 3) and dark period (*n* = 6) of the 24 h light cycle (Marques et al., 2011b). The genes selected for analysis included Angiotensin receptor 1a (*Agtr1a*), Angiotensin receptor 1b (*Agtr1b*), Angiotensin receptor 2 (*Agtr2*), Mas receptor (*Mas1*), Angiotensinogen (*Agt*), Renin 2 (*Ren2*), Angiotensin converting enzyme (*Ace*), and Angiotensin converting enzyme 2 (*Ace2*). We performed a whole-genome analysis using the GEO tools, including false discovery rate <0.05, to determine whether genes were differentially expressed in BPN/3J vs. BPH/2J mice at a given period (light and dark) as well as differences between light vs. dark in each strain.

### Statistical analysis

Cardiovascular and gene array data were expressed as mean ± standard error of the mean (SEM). The acute drug responses compared the average change during the 15–30 min period post injection with the 30 min control period immediately prior to injection. Effect of treatment (“*treat*”) was a within animal analysis which represents the response compared with baseline. Effect of drug (“*drug”*) represents the response to a drug compared with the response to vehicle (between animal comparisons). The between groups sums of squares was partitioned into main effects of drug (drug compared with vehicle—between animal analysis), strain (BPH/2J vs. BPN/3J), and their interaction (drug × strain). In the chronic drug treatment study, a within animal analysis was used to compare either the 24 h average baseline or 12 h (light or dark period) with the corresponding values following 7 days of each treatment (SC and ICV). Effect of route (“*route*”) was a within animal analysis of the average change from baseline in SC compared with ICV treatment. The data were analyzed by multi-factor split-plot analysis of variance (ANOVA), which allowed for within animal and between animal contrasts (Snedecor and Cochran, 1980). A combined residual was used that pooled the between and within animal variance as described previously (Korner et al., 1987). A probability of *P* < 0.05 was considered significant.

## Results

### Basal BP, HR, and locomotor activity during dark and light periods

**Figure 1:**
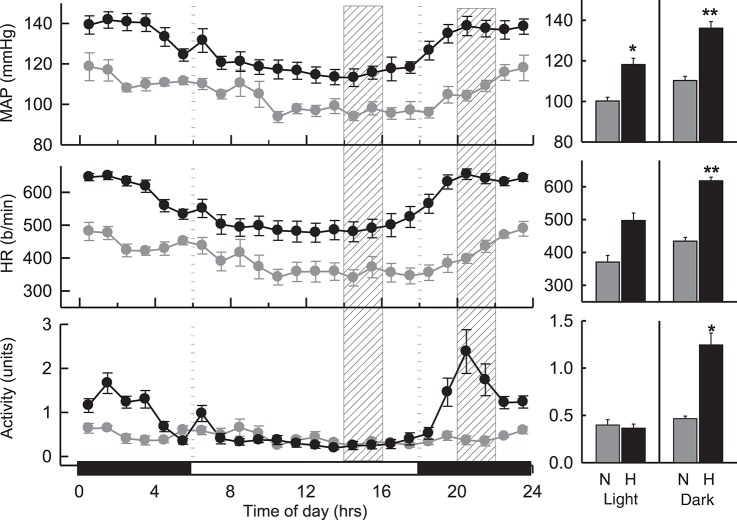
Hourly averaged data showing the circadian variation of MAP (mmHg), HR (beats/min) and activity (units) during the dark (active) (outer panels) and light (inactive) (Middle) phases in BPN/3J (*n* = 7) and BPH/2J mice (*n* = 8). Shaded columns represent time-range of conduct of light (inactive) experiments **(Left)** and dark (active) experiments **(Right)**. Bar graphs on right represent average MAP, HR, and locomotor activity during the light (inactive) and dark (active) periods in BPN/3J (N) and BPH/2J (H) mice. Values are mean±SEM. For comparisons between strains across the 12 hr light or dark period. ^*^P < 0.05; ^**^P < 0.01.

MAP in hypertensive BPH/2J mice (*n* = 8) was greater than that observed in BPN/3J mice (*n* = 7) over a 24 h period (*P* < 0.001; Figure 1). During the dark (active) period, MAP was 23% greater (*P*_strain_ = 0.002), HR was 42% greater (*P*_strain_ = 0.009), and locomotor activity was 2.7-fold greater (*P*_strain_ = 0.005) in BPH/2J mice compared with BPN/3J mice. During the light (inactive) period, MAP in BPH/2J mice was 18% greater than in BPN/3J mice (*P*_strain_ = 0.01) and HR tended to be higher in BPH/2J mice (*P*_strain_ = 0.05) but activity was comparable between strains (*P*_strain_ = 0.9).

### Effect of acute ICV treatments on cardiovascular measurements

#### Vehicle and sham (dark period)

**Figure 2:**
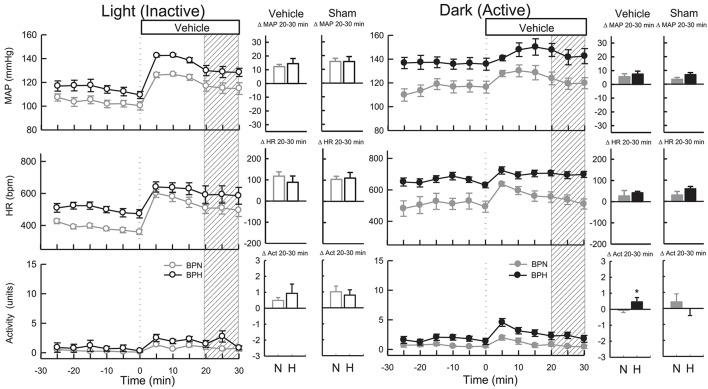
Line graphs represent MAP, HR, and locomotor activity responses to vehicle during the inactive period **(Left)** and active period **(Right)** between BPN/3J (*n* = 6–8, gray) and BPH/2J (*n* = 4–8, black) mice. Each dot represents the mean value averaged across a 5-min period. The dashed vertical reference line represents the time-point of administration of treatment. Shaded area represents the period analyzed for effect of treatment. Bar graphs represent average change in MAP, HR, and locomotor activity induced by vehicle and sham treatment (*n* = 5/strain) during the inactive period (Middle), and active period **(Right)** in BPN/3J (N, gray) and BPH/2J mice (H, black). Deltas represent the difference between the 30-min control period and 20–30 min post-injection. ^*^ *P* < 0.5.

As ICV injections involved handling and disturbing the conscious mice, a vehicle control treatment was included from which the dark (active) drug treatments could be compared. All vehicles (Ringer's solution, DMSO, and Na_2_CO_3_) produced comparable BP and HR responses (*P* > 0.6 for all) during the dark period. As such these measurements were pooled for analysis. Microinjection of vehicle resulted in a small pressor response in BPN/3J (*n* = 8, 5 ± 2 mmHg, *P*_*treat*_ = 0.009) and BPH/2J mice (*n* = 8, 7 ± 2 mmHg, *P*_*treat*_ < 0.001), which were similar between strains (*P*_strain_ = 0.4, Figure 2). Following treatment with vehicle, HR was elevated in BPH/2J mice (*P*_treat_ < 0.001) but not BPN/3J mice (*P*_treat_ = 0.3), yet HR responses were similar between strains (*P*_strain_ = 0.3). Locomotor activity following vehicle administration was reduced in BPN/3J mice (*P*_treat_ = 0.05) but comparable with baseline in BPH/2J mice (*P*_treat_ = 0.2, *P*_strain_ = 0.04, Figure 2). Sham injections (*n* = 5/strain) during the dark period caused similar effects on BP, HR, and locomotor activity compared with vehicle injections in each strain (*P* > 0.1 for all).

#### Vehicle and sham (light period)

All vehicles (Ringer's solution, DMSO, and Na_2_CO_3_) produced comparable BP and HR responses (*P* > 0.7 for all) during the light period, so measurements were pooled for analysis. Vehicle microinjections resulted in pressor responses in BPN/3J (*n* = 6, 13 ± 2 mmHg, *P*_treat_ < 0.001) and BPH/2J mice (*n* = 4, 16 ± 3 mmHg, *P*_treat_ < 0.001), which were of similar magnitude between strains (*P*_strain_ = 0.2, Figure 2). Vehicle treatment increased HR comparably in both strains (*P*_treat_ < 0.001 both, *P*_strain_ = 0.3). Locomotor activity was elevated in BPN/3J (*P*_treat_ < 0.001) and BPH/2J mice (*P*_treat_ = 0.01) and this response was similar in both strains (*P*_strain_ = 0.42, Figure 2). Sham injections (*n* = 5/strain) during the light period caused similar effects on BP, HR, and locomotor activity compared with vehicle injections in each strain (*P* > 0.1 for all) demonstrating that the injection procedure rather than administration of vehicle caused the moderate pressor and tachycardic effects.

#### Candesartan (dark period)

**Figure 3:**
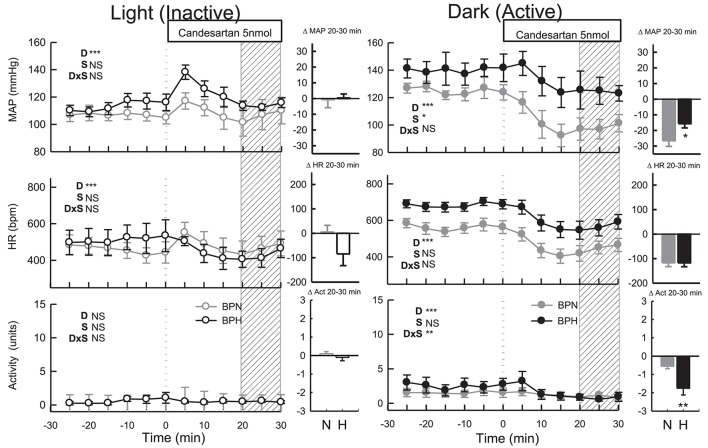
Line graphs represent MAP, HR, and locomotor activity responses to candesartan (5 nmol) during the inactive period **(Left)** and active period **(Right)** in BPN/3J (*n* = 6–8, gray) and BPH/2J (*n* = 4–8, black) mice. Each dot represents the mean value averaged across a 5-min period. The dashed vertical reference line represents the time-point of administration of treatment. Shaded area represents the period analyzed for effect of treatment. Bar graphs represent average change in MAP, HR, and locomotor activity induced by treatment during the inactive period (Middle), and active period **(Right)** in BPN/3J (N, gray) and BPH/2J mice (H, black). Deltas represent the difference between the 30-min control period and 20–30 min post-injection. Effect of drug (D) compared with vehicle, effect of strain (S) and drug by strain interaction (DxS) are shown at the top left of each line graph. Values are mean ± SEM; ^*^P < 0.05; ^**^P < 0.01; ^***^P < 0.001.

ICV administration of candesartan reduced MAP in both strains (*P*_treat_ < 0.001) but depressor responses in BPN/3J mice (*n* = 8, −27 ± 4 mmHg) were 1.7-fold greater than BPH/2J mice (*n* = 8, −16 ± 3 mmHg, *P*_strain_ = 0.03, Figure 3). In comparison with the response induced by vehicle, the effective reduction in MAP to candesartan was 31 mmHg in BPN/3J mice (*P*_drug_ < 0.001) and 23 mmHg in BPH/2J mice (*P*_drug_ < 0.001, Figure 3). Candesartan injected ICV reduced HR similarly in BPN/3J (−117 ± 15 mmHg, *P*_treat_ < 0.001) and BPH/2J mice (−118 ± 15 bpm, *P*_treat_ < 0.001; *P*_strain_ = 1.0) and reduced locomotor activity in both strains (*P*_*treat*_ < 0.01 both) but more so in BPH/2J mice (*P*_strain_ < 0.001).

#### Candesartan (light period)

Following candesartan administration, MAP returned to baseline levels in BPN/3J (*n* = 6, *P*_treat_ = 0.8) and BPH/2J mice (*n* = 4, *P*_treat_ = 0.9, *P*_strain_ = 0.9, Figure 3). Compared with the moderate elevation in BP following vehicle, the effective reduction in MAP to candesartan was 13 mmHg in BPN/3J (*P*_drug_ = 0.002) and 16 mmHg in BPH/2J mice (*P*_drug_ = 0.002, Figure 3). Candesartan treatment also reduced HR in BPH/2J mice (*P*_treat_ = 0.04) but not in BPN/3J mice (*P*_treat_ = 0.8, *P*_strain_ = 0.03, Figure 3) and locomotor activity was unaffected in both strains (*P*_treat_ > 0.5).

#### AngII (dark period)

ICV administration of AngII induced pressor responses which were comparable in BPN/3J mice (*n* = 4, 38 ± 2 mmHg) and BPH/2J mice (*n* = 5, 34 ± 1 mmHg, *P* = 0.15). Tachycardic responses were greater in BPN/3J mice (BPN/3J: 289 ± 9 bpm; BPH/2J: 147 ± 8 bpm, *P* < 0.001) whereas the locomotor activity responses were greater in BPH/2J mice (BPN/3J: 1.0 ± 0.2 units; BPH/2J: 2.1 ± 0.3 units, *P* = 0.001).

#### Tempol (dark period)

**Figure 4:**
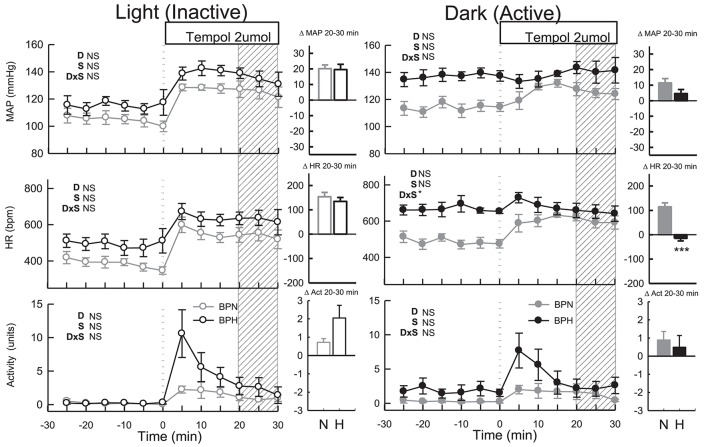
Line graphs represent MAP, HR, and locomotor activity responses to tempol (2 μmol) during the inactive period **(Left)** and active period **(Right)** between BPN/3J (*n* = 6, gray) and BPH/2J (*n* = 4–6, black) mice. Each dot represents the mean value averaged across a 5-min period. The dashed vertical reference line represents the time-point of administration of treatment. Shaded area represents the period analyzed for effect of treatment. Bar graphs represent average change in MAP, HR, and locomotor activity induced by treatment during the inactive period (Middle), and active period **(Right)** in BPN/3J (N, gray) and BPH/2J mice (H, black). Deltas represent the difference between the 30-min control period and 20–30 min post-injection. Effect of drug (D) compared with vehicle, effect of strain (S) and drug by strain interaction (DxS) are shown at the top left of each line graph. Values are mean ± SEM; ^*^P < 0.05; ^***^P < 0.001.

ICV tempol elevated MAP (*P* < 0.001), HR (*P* < 0.001) and locomotor activity in BPN/3J (*n* = 6, *P*_treat_ = 0.02, Figure 4) but had little effect in BPH/2J mice (*n* = 6) since MAP, HR, and locomotor activity returned to levels comparable with baseline (*P*_treat_ > 0.2 all, Figure 4). The small effects of tempol were comparable between strain (*P*_strain_ = 0.08) and were similar to those produced by vehicle in both strains (*P*_drug_ > 0.4).

#### Tempol (light period)

Administration of tempol increased MAP, HR and locomotor activity similarly in both strains (*P*_treat_ < 0.05, *P*_strain_ > 0.1, Figure 4). In BPN/3J mice (*n* = 6) the pressor response induced by tempol was greater than that of vehicle treatment (*P*_drug_ = 0.03, Figure 4). However, pressor responses following tempol and vehicle treatment were similar in BPH/2J mice (*n* = 4, *P*_drug_ = 0.4).

#### Resveratrol (dark period)

**Figure 5:**
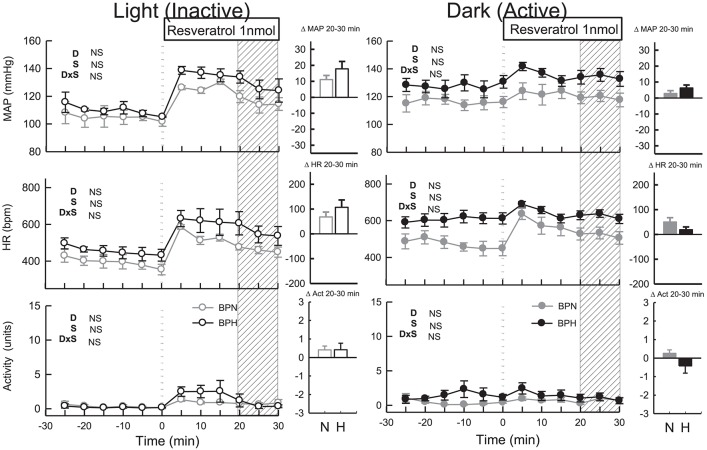
Line graphs represent MAP, HR, and locomotor activity responses to resveratrol (1 nmol) during the inactive period **(Left)** and active period **(Right)** between BPN/3J (*n* = 4–6, gray) and BPH/2J (*n* = 4–7, black) mice. Each dot represents the mean value averaged across a 5-min period. The dashed vertical reference line represents the time-point of administration of treatment. Shaded area represents the period analyzed for effect of treatment. Bar graphs represent average change in MAP, HR, and locomotor activity induced by treatment during the inactive period (Middle), and active period **(Right)** in BPN/3J (N, gray) and BPH/2J mice (H, black). Deltas represent the difference between the 30-min control period and 20–30 min post-injection. Effect of drug (D) compared with vehicle, effect of strain (S) and drug by strain interaction (DxS) are shown at the top left of each line graph. Values are mean ± SEM.

MAP was slightly elevated in BPH/2J mice following microinjection of resveratrol (*n* = 7, *P*_treat_ = 0.003, Figure 5). However, MAP responses to resveratrol were similar to responses induced by vehicle treatment in both BPN/3J (*n* = 6) and BPH/2J mice (*P*_drug_ > 0.6). HR responses following resveratrol administration were also similar to vehicle responses in BPN/3J and BPH/2J mice (*P*_drug_ > 0.2) and there was little effect on locomotor activity in both strains (*P*_treat_ = 0.2 both, *P*_strain_ = 0.1, Figure 5).

#### Resveratrol (light period)

Following resveratrol treatment MAP was elevated to a similar extent in both strains (*n* = 4/strain, *P*_treat_ < 0.001, *P*_strain_ = 0.1, Figure 5). However, the pressor responses induced by resveratrol were similar to responses induced by vehicle in both strains (*P*_drug_ = 0.7 both). HR responses to resveratrol microinjection were also similar to vehicle responses in both strains (*P*_drug_ > 0.2 both) as was locomotor activity (*P*_drug_ > 0.5).

### Effect of ICV treatments on cardiovascular response to 5-min restraint stress

**Figure 6:**
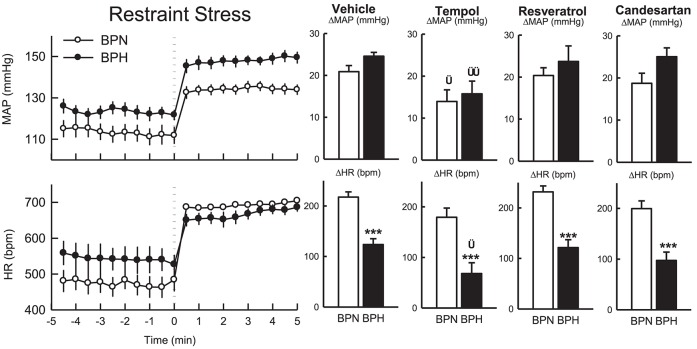
Line graphs represent the MAP and HR responses in BPN/3J mice (white circles, *n* = 5–6) and BPH/2J mice (black circles; *n* = 4) before and during restraint stress. Each dot represents mean value; averaged across a 30-s period. Bar graphs represent average changes in MAP and HR, and locomotor activity in BPN/3J (white bar) and BPH/2J (black bar) in response to stress following vehicle, tempol, resveratrol, and candesartan treatment. The average responses were calculated over 5-min of stress exposure and a 5-min control period in each animal. Values are mean ± SEM. Comparison of BPH/2J with BPN/3J mice represented by ^***^P < 0.001. Comparison of response to drug compared with vehicle in each strain is represented by ^†^*P* < 0.05; ^††^*P* < 0.01.

Following vehicle microinjections the pressor response induced by restraint stress tended to be greater in BPH/2J (*n* = 4, 25 ± 1 mmHg) than BPN/3J mice (*n* = 6, 21 ± 1 mmHg, *P*_strain_ = 0.07, Figure 6), whereas the tachycardic response was greater in BPN/3J compared with BPH/2J mice (*P* < 0.001). Locomotor activity was suppressed by restraint (data not shown).

In comparison to vehicle, the pressor responses induced by restraint stress following treatment with tempol were reduced by 33% in BPN/3J (*n* = 6) and 36% in BPH/2J mice (*n* = 4, *P*_drug_ = 0.01 both, *P*_strain_ = 0.6, Figure 6). The increase in HR induced by restraint was attenuated in BPH/2J mice following tempol microinjection (*P*_drug_ = 0.03) and also tended to be reduced in BPN/3J mice (*P*_drug_ = 0.07) compared with the response following vehicle.

Resveratrol had little effect on the pressor or tachycardic response to restraint stress in either BPN/3J (*n* = 5) of BPH/2J (*n* = 4) when compared with responses following vehicle (*P*_drug_ > 0.5, Figure 6).

Pressor and tachycardic responses induced by restraint stress following candesartan administration were comparable with responses following vehicle treatment in BPN/3J (*n* = 6) and BPH/2J mice (*n* = 4, *P*_drug_ > 0.3, Figure 6).

## Effect of chronic central administration of losartan on cardiovascular and locomotor measurements

### Preliminary ICV dose response to losartan

There was a small depressor response to the ICV bolus of losartan delivered at a dose of 0.4 μg in BPH/3J mice (*P* = 0.03) but the response in BPN/3J mice was comparable with vehicle (*P* = 0.2). In contrast to vehicle, doses of 2 and 10 μg of losartan reduced BP in both strains (*P* < 0.03). The BP response to 10 μg of losartan lasted approximately 60 min and tended to be different to the response to 10 μg of losartan delivered i.p. in both strains (*P* < 0.06). Consequently a dose of 10 μg/h (22 nmol/h) was selected for the chronic infusion study.

### Main chronic study

**Figure 7:**
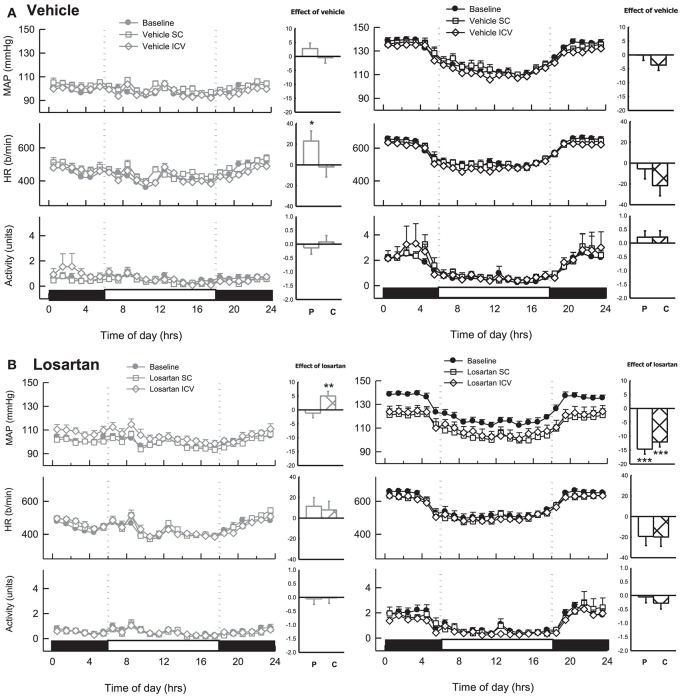
Line graphs represent hourly averages of mean arterial pressure (MAP), heart rate (HR, beats per minute) and activity over a 24 h period highlighting the dark (active) and light (inactive) phases. BPN/3J (BPN, gray, left) and BPH/2J mice (BPH, black, right) were chronically infused with **(A)** vehicle (*n* = 6/strain) or **(B)** Losartan (*n* = 7–8/strain, 22 nmol/h). MAP, HR and activity were measured at baseline (filled circles) during SC treatment (unfilled squares) and ICV treatment (unfilled diamonds). Histograms represent mean difference ± SED from baseline following peripheral SC infusion of treatment (P, unfilled bars) and central ICV infusion of treatment (C, hatched bars) ^*^P < 0.05; ^**^P < 0.01; ^***^P < 0.001 for the probability based on ANOVA.

During baseline measurements, MAP, HR and activity were greater in BPH/2J (*n* = 13) compared with BPN/3J mice (*n* = 14, *P* < 0.01). Neither ICV nor SC infused vehicle changed MAP or locomotor activity in either BPN/3J (*n* = 6) or BPH/2J mice (*n* = 6, *P*_treat_ > 0.1, Figure 7A). There was minimal effect of ICV or SC vehicle on HR in both strains (*P*_treat_ > 0.06), except for a modest effect of SC vehicle infusion on HR in BPN/3J mice (*P*_treat_ = 0.04, Figure 7A). ICV infusion of losartan (22 nmol/h) lowered MAP in BPH/2J mice (*n* = 7, −12.1 ± 1.8 mmHg, *P*_treat_ < 0.001, Figure 7B). However, SC infused losartan (22 nmol/h) also lowered MAP in BPH/2J (−14.7 ± 1.8 mmHg, *P*_treat_ < 0.001) and the effect of route (ICV vs. SC) was comparable (*P* = 0.50). Losartan infused SC had no effect on MAP in BPN/3J mice (*n* = 8, *P*_treat_ = 0.99). Central losartan treatment induced a mild increase in MAP (+5.0 ± 1.7 mmHg, *P*_treat_ = 0.008). However, the effect of losartan on MAP in BPN/3J mice was comparable with vehicle whether delivered SC or ICV (*P*_interaction_ > 0.23). ICV and SC infusion of losartan had minimal effect on HR and locomotor activity in BPN/3J or BPH/2J mice compared with baseline (*P* > 0.07 for all).

### Within strain differences in effect of drugs on map during the light and dark period

**Figure 8:**
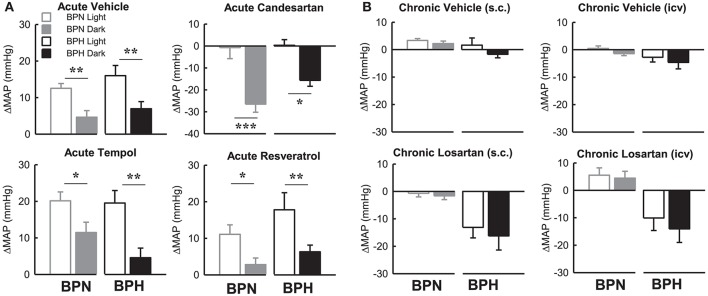
Bar graphs represent average changes in MAP in BPN/3J (gray bars) and BPH/2J (black bars) during the light period (unfilled) and dark period (filled) in response to **(A)** bolus ICV administration of Vehicle (*n* = 4–8), Candesartan (*n* = 4–8), Tempol (*n* = 4–6) and Resveratrol (*n* = 4–7) and **(B)** following 1 week infusion of Vehicle (s.c.) (*n* = 6/strain), Vehicle (ICV) (*n* = 6/strain), Losartan (s.c.) (*n* = 7–8/strain) and Losartan (ICV)(*n* = 7–8/strain). The average responses in **(A)** were calculated over the 15–30-min period post ICV injection compared with a 30-min control period in each animal. The average response in **(B)** were calculated over the 12 h light and 12 h dark periods compared with baseline pre-treatment values in each animal. Values are mean ± SEM. Comparison of the response in a given strain during the light compared with the dark period is represented by ^*^P < 0.05; ^**^P < 0.01; ^***^P < 0.001.

Candesartan produced a depressor response in both strains only during the dark period which was markedly different compared with the lack of response from baseline during the light period in both BPN/3J (*P* < 0.001) and BPH/2J mice (*P* < 0.05, Figure 8A). The pressor responses following vehicle, tempol, and resveratrol were all greater in the light period compared with the dark period in both BPN/3J and BPH/2J mice (*P* < 0.05, Figure 8). Interestingly there was no difference between the light compared with the dark period in the effect of chronic vehicle or losartan (ICV or S.C.) on MAP in BPN/3J or BPH/2J mice (*P* > 0.12, Figure 8B).

### Effect of chronic administration of losartan on cardiovascular response to stress

#### 1 h of restraint stress

**Figure 9:**
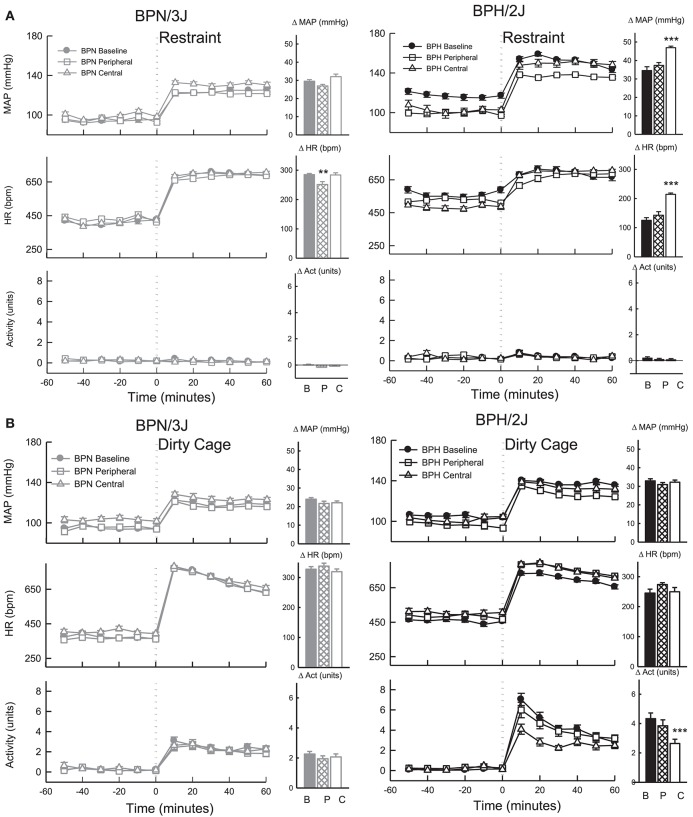
Line graphs represent average mean arterial pressure (MAP), heart rate (HR, beats per minute) and locomotor activity responses before and during **(A)** restraint stress and **(B)** Dirty cage switch stress in BPN/3J (left panels, gray, *n* = 8) and BPH/2J (right panels, black, *n* = 6) mice. Responses to stress were measured during pre-treatment baseline (closed circles) and during peripheral losartan (open squares) and central losartan (open triangles). Each dot represents mean ± SEM, averaged across a 10 min period. Bar graphs represent average change in MAP, HR and locomotor activity in response to stress during baseline (filled bars) and peripheral losartan (hashed bars) and central losartan (unfilled bars) treatment. Values are mean difference ± SED and compares treatment response to baseline in each strain ^**^P < 0.01; ^***^P < 0.001. Legend: B, Baseline, P, Peripheral losartan; C, Central losartan.

The pressor response induced by 1 h of restraint stress was greater in BPH/2J mice (*n* = 6) than BPN/3J mice (*n* = 8, *P* < 0.05) whereas the tachycardic response was greater in BPN/3J mice (*P* < 0.001). Infusion of losartan SC for 7 days did not influence the pressor response to restraint in either strain (*P* > 0.7) but did attenuate the tachycardic response to this stress in BPN/3J mice (*P* < 0.005, Figure 9). ICV infusion of losartan augmented the pressor and tachycardic response to restraint in BPH/2J mice (*P* < 0.001) but not BPN/3J (*P* > 0.3).

#### 1 h dirty cage switch stress

The pressor response induced by dirty cage switch stress was greater in BPH/2J (*n* = 6) than BPN/3J mice (*n* = 8, *P* < 0.001) whereas the tachycardic response was much greater in BPN/3J mice (*P* < 0.001). ICV and SC losartan treatment had minimal effect on the pressor and tachycardic response to dirty cage switch stress in BPN/3J and BPH/2J mice (*P* > 0.6, Figure 9). Losartan infused SC had no influence on the surge in locomotor activity induced by dirty cage switch stress in both strains (*P* > 0.4). However, ICV losartan attenuated the locomotor activity response induced by dirty cage switch stress specifically in BPH/2J mice (*P* < 0.001).

#### Gene array

*Agt* mRNA was greater in hypothalamic tissue collected during the dark period compared with the light period in both BPN/3J and BPH/2J mice (*P* < 0.01).

**Figure 10:**
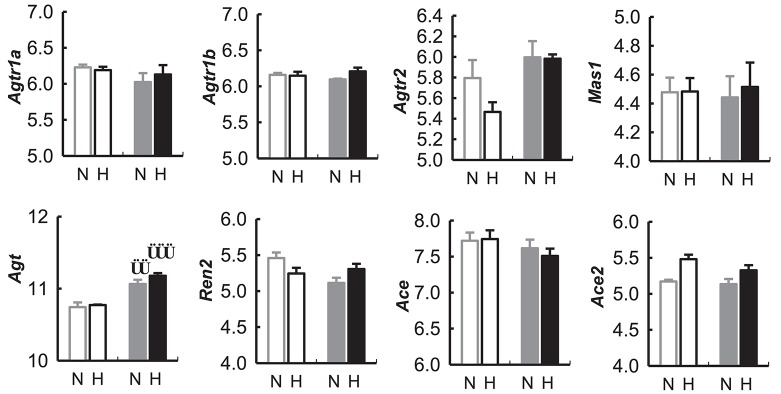
Expression of genes (measured as mRNA) in the renin-angiotensin related to the renin-angiotensin system in the hypothalamus of BPN/3J mice (N, Gray) and BPH/2J mice (H, Black) during the light (unfilled bars) and dark periods (filled bars) of the 24 h cycle, from gene array data obtained from the Gene Expression Omnibus Repository (Marques et al., 2011b). Angiotensin receptor 1a (*Agtr1a*), Angiotensin receptor 1b (*Agtr1b*), Angiotensin receptor 2 (*Agtr2*), Mas receptor (*Mas1*), Angiotensinogen (*Agt*), Renin 2 (*Ren2*), Angiotensin converting enzyme (*Ace*), Angiotensin converting enzyme 2 (*Ace2*). Comparison of mRNA abundance during the light vs. dark period in a given strain ^††^*P* < 0.01; ^†††^*P* < 0.001.

There was no difference between BPN/3J and BPH/2J mice in any of the other mRNA assessed (i.e., *Agtr1a, Agtr1b, Agtr2, Mas1, Agt, Ren2, Ace, Ace2*) from hypothalamic tissue collected during the dark or light period of the 24 h light cycle (Figure 10).

## Discussion

The major finding of the present study was that acute ICV administration of AT_1_ receptor antagonist did not cause greater depressor responses in BPH/2J mice compared with BPN/3J mice. ICV infusion of losartan for over 7 days had little effect on BP in BPN/3J mice but induced a moderate decrease in BP in BPH/2J mice. The hypotensive effect of ICV losartan in BPH/2J mice was comparable with that observed following systemic administration. Whilst it is possible that the peripheral dose of losartan crossed the BBB to produce a hypotensive effect, it is more likely that the central dose leaked into the periphery to block peripherally located AT_1_ receptors. Additionally, acute ICV administration of tempol or resveratrol had no major influence on BP in either strain. Collectively, the apparent lack of effect on BP of acute and steady state inhibition of central AT_1_ receptors and acute ROS inhibition, suggests that overactivity of central AT_1_ receptor or associated ROS signaling are unlikely to be contributing to high BP in this model of hypertension.

### Role of the central RAS and ROS in BPH/2J hypertension

The primary evidence that there is not a contribution from central AT_1_ receptors overactivity to the hypertension in BPH/2J mice is based on the equal or smaller depressor response to acute central AT_1_ receptor inhibition in BPH/2J compared with BPN/3J mice. Bunting and colleagues also report a similar lack of contribution of central AT_1_ receptors to the hypertension in SHR on a normal salt diet (Bunting and Widdop, 1995). Since acute bolus ICV administration of AT_1_ receptor antagonists produce only transient effects on BP, we also sought to determine the effect of steady state inhibition of central AT_1_ receptors by continuously infusing losartan ICV for 1 week. In an attempt to distinguish between the peripheral and central effects of losartan, we compared the BP effects following central (ICV) and peripheral (SC) administration of the same dose. Our results show that despite losartan producing a greater hypotensive effect in BPH/2J mice than BPN/3J mice, the effect was comparable whether it was delivered ICV or SC. Based on these results it is unknown whether peripherally circulating losartan may also inhibit central AT_1_ receptors either via actions at circumventricular organs or even behind the BBB. Indeed, whilst some studies suggest that losartan does not readily cross the BBB (Wong et al., 1990; Bui et al., 1992), other studies show evidence that losartan, or its active metabolite EX3174 can cross the BBB, bind to AT_1_ receptors and inhibit the functional effects of central AngII in sites that regulate BP, including the NTS and PVN (Song et al., 1991; Li et al., 1993; Santos et al., 1995). The differences reported in the ability of losartan or its metabolites to cross the BBB, could be dependent on factors including the dose, duration or even the route administration. However, a more likely explanation for the comparable hypotensive effect of SC and ICV losartan, is that the hypotensive effect of ICV losartan in BPH/2J mice was due to the actions of losartan which leaked into the periphery. Indeed, since CSF drains into the lymph, compounds delivered into the CSF inevitably make their way into the plasma (Boulton et al., 1997). However, if we consider the volume of distribution via the two different routes of administration, it is clear that the relative drug level reached in the brain would be far greater following local ICV delivery of losartan compared with via SC administered losartan. Thus, regardless of leakage into the periphery, if there was indeed a greater contribution of the central AT_1_ receptors to hypertension in BPH/2J mice, we would see a greater hypotensive effect following ICV administration than following SC administration. This type of scenario was reported by Huang and colleagues, where chronic ICV administration of losartan produced greater hypotensive effects in SHR with salt induced hypertension compared with intravenous administration of the same dose, suggesting that chronic central AT_1_ receptor inhibition can reveal a greater contribution of central AT_1_ receptor activity if it is apparent (Huang and Leenen, 1996). Furthermore our results are much like those reported by two separate studies by Kawano and Bunting and colleagues, where the hypotensive effect of chronic central AT_1_ receptor inhibition in SHR was shown to be due to the effect of peripheral rather than central AT_1_ receptor inhibition (Kawano et al., 1994; Bunting and Widdop, 1995). Finally if we consider both the acute and chronic findings, it is apparent that the greater hypotensive effect of chronic ICV losartan in BPH/2J than BPN/3J mice is in direct contrast to the effect of acute central AT_1_ receptor inhibition, which is smaller in BPH/2J mice. Actually, the BP response to chronic ICV infusion of losartan is more consistent with the effect of peripheral RAS blockade in BPH/2J mice, previously determined using the ACE inhibitor enalaprilat which does not readily cross the BBB (Jackson et al., 2013). Thus if the hypotensive effect following both s.c. and ICV infused losartan is due to blockade of peripheral AT_1_ receptors, then we can conclude that there is no greater contribution of central AT1 receptors to the hypertension in BPH/2J compared with BPN/3J mice. Nonetheless, the present study is limited by the fact that it is unclear the degree to which the peripheral (SC) dose of losartan crossed the BBB to contribute to the hypotensive effect in BPH/2J mice.

In addition to the pharmacological assessment of the role of the central RAS in BPH/2J mice our analysis of the results of gene expression in the hypothalamus of BPH/2J mice (Marques et al., 2011b) suggests no differences in the expression of the angiotensin converting enzymes (ACE and ACE2), angiotensin receptors (AT_1A_, AT_1B_, AT_2_, and Mas receptors) or angiotensinogen in the hypothalamus of BPH/2J and BPN/3J strains (Marques et al., 2011b). Furthermore, the pressor response to ICV AngII administration was comparable in BPN/3J and BPH/2J mice, suggesting that the central AngII signaling pathway does not appear to be abnormal in BPH/2J mice. Taken together with the lesser effect of acute AT_1_R inhibition, it appears that the role of the central RAS is minimal in this model of hypertension.

ROS have also been demonstrated to contribute to elevated BP in hypertensive animal models where a greater contribution of the central RAS is apparent, including AngII induced hypertension (Zimmerman et al., 2004; Campese et al., 2005), Dahl salt sensitive hypertensive rats (Fujita et al., 2007) and high salt diet induced hypertension in SHR (Koga et al., 2008). This association between the central RAS and ROS involves activation of AT_1_ receptors resulting in NADPH oxidase (NOX) activation and superoxide production (Zimmerman et al., 2002; Chan et al., 2005). Since the mechanism mediating hypertension in BPH/2J mice appears to be independent of central AT_1_ receptor activity, it is not surprising that there was minimal effect on BP following treatment with the SOD mimetic tempol or resveratrol which has ROS scavenging properties (Leonard et al., 2003). Thus, our acute results suggest that oxidative stress in the CNS is not likely to be a major contributor to BP maintenance or hypertension in BPH/2J mice. However, due to potential differences in the effect of acute and chronic central inhibition of ROS, it would be important in future to validate these acute findings by using chronic central inhibition as well.

### Role of central RAS and ROS in circadian and stress related changes in BP

The present findings reveal clear circadian related differences in the influence of central AT_1_ receptors on BP maintenance based on the marked depressor response to acute AT_1_ receptor inhibition during the dark period compared with the minimal effect on BP during the light period of the 24 h light cycle. Indeed, analysis of gene expression in the hypothalamus shows whilst there is little difference in the hypothalamic expression of AT receptors from light to dark period, there is an elevation in the expression of angiotensinogen during the dark period in both strains, which likely results in greater AngII production during this period, although AngII protein levels would need to be measured in future to confirm this suggestion. Interestingly the analysis of the effect of chronic losartan (ICV or SC) does not reveal a difference in magnitude of the hypotensive effect during the 12 h dark compared with the 12 h light period. This is constant with our previous study with a larger systemic oral dose of losartan which was equally effective during the dark and light period (Palma-Rigo et al., 2011). In the present study it was also apparent that the pressor response to resveratrol and tempol were lower in the dark compared with the light period but this was also apparent in vehicle treated mice. This finding is likely related to the pressor response (associated with handling for injections) being minimized by a ceiling effect during the dark period since baseline BP is higher during the dark period compared with the light.

The central RAS is a well-recognized regulator of stress, acting within limbic, hypothalamic and medullary brain regions critical for the manifestation of the cardiovascular response to aversive stressors (Mayorov, 2007; Chen et al., 2009). In the present study we assessed the effect of acute ICV AT_1_ receptor and ROS inhibition on the 5 min pressor response to restraint stress, as we have previously shown this response to be predominantly mediated by the SNS. However, acute central AT_1_ receptor inhibition in the present study had no effect on the pressor response to 5 min restraint stress in either strain, suggesting that they are not crucially involved in the initial sympathetically mediated pressor response to stress. Previous studies show that central AT_1_ receptors particularly those in the RVLM are more important in the maintenance of the pressor response over the course of an hour than the first 5 min of the stress (Chen et al., 2012), thus we assessed the response to 1 h stress exposure in chronic losartan treated mice. However, chronic central AT_1_ receptor inhibition with losartan did not inhibit the 1 h pressor response to restraint or dirty cage swap stress in either strain, which is contrary to expectation as central administration of AT_1_ receptor antagonists usually reduce pressor responses to stress. Interestingly, centrally but not peripherally administered losartan actually augmented the pressor response to restraint stress in BPH/2J mice, suggesting an abnormal inhibitory effect of the central RAS in BPH/2J mice.

The present findings also show tempol administration attenuated the pressor response induced by restraint stress to a similar extent in both strains. Whilst these findings suggest that ROS are not contributing to the exaggerated stress response reported in BPH/2J mice (Davern et al., 2010) they do demonstrate that central ROS plays a role in mediating the normal cardiovascular response to stress. This finding is consistent with an influence of ROS in central autonomic pressor pathways known to influence stress (Mayorov et al., 2004; De Matteo et al., 2006). It has been suggested that AT_1_ receptor-ROS signaling is responsible for producing the pressor response to stress mediated by central autonomic pressor pathways (Mayorov et al., 2004). However, since acute and chronic AT_1_ receptor blockade did not attenuate the pressor response to the short or longer duration stressors, the influence of ROS on the cardiovascular response to stress shown in the present study appears to be independent of AT_1_ receptor signaling.

## Conclusion

In conclusion, our acute pharmacological studies demonstrate that central AT_1_ receptor activity is crucial for normal regulation of BP specifically during the dark (active) period but less so during the light (inactive) period, whilst central ROS seem to be more important in controlling the cardiovascular response to stress. Furthermore, whilst there is some degree of uncertainty as to whether the chronic peripheral dose of losartan has central effects, the fact that the ICV dose does not produce a greater hypotensive effect compared with the SC dose suggests that greater central AT_1_ receptor activity does not contribute to the hypertension in BPH/2J mice. This is supported by the lesser hypotensive response to acute AT_1_ receptor inhibition in BPH/2J mice and taken together our results show little evidence of a contribution of central AT_1_ receptors to hypertension in BPH/2J mice. This apparent lack of contribution of central RAS in this model contrasts the well-recognized influence of central AT_1_ receptor activity in other forms of hypertension such as DOCA-salt hypertension, salt induced hypertension in SHR and cold induced hypertension (Huang and Leenen, 1996; Park and Leenen, 2001; Sun et al., 2002), but is consistent with a lack of contribution reported in SHR on a normal salt diet (Kawano et al., 1994; Bunting and Widdop, 1995). Nonetheless, the greater hypotensive effect of chronic AT_1_ receptor inhibition in the periphery of BPH/2J compared with BPN/3J mice, validates our recent findings based on acute ACE inhibition (Jackson et al., 2013), which suggest that greater peripheral RAS activity plays an important role in the hypertension in BPH/2J mice.

## Author contributions

KJ, FM, KL, PD, and GH performed the study including experimental data collection and analysis, preparation, and writing and editing of the manuscript.

### Conflict of interest statement

The authors declare that the research was conducted in the absence of any commercial or financial relationships that could be construed as a potential conflict of interest.
